# Correction: Evaluation of kidney function among people living with HIV initiating antiretroviral therapy in Zambia

**DOI:** 10.1371/journal.pgph.0001957

**Published:** 2023-05-16

**Authors:** Jake M. Pry, Michael J. Vinikoor, Carolyn Bolton Moore, Monika Roy, Aaloke Mody, Izukanji Sikazwe, Anjali Sharma, Belinda Chihota, Miquel Duran-Frigola, Harriet Daultrey, Jacob Mutale, Andrew D. Kerkhoff, Elvin H. Geng, Brad H. Pollock, Jaime H. Vera

The captions for Figs 4 and 5 are incorrect. Please see below the correct captions and figures.

**Fig 4:**
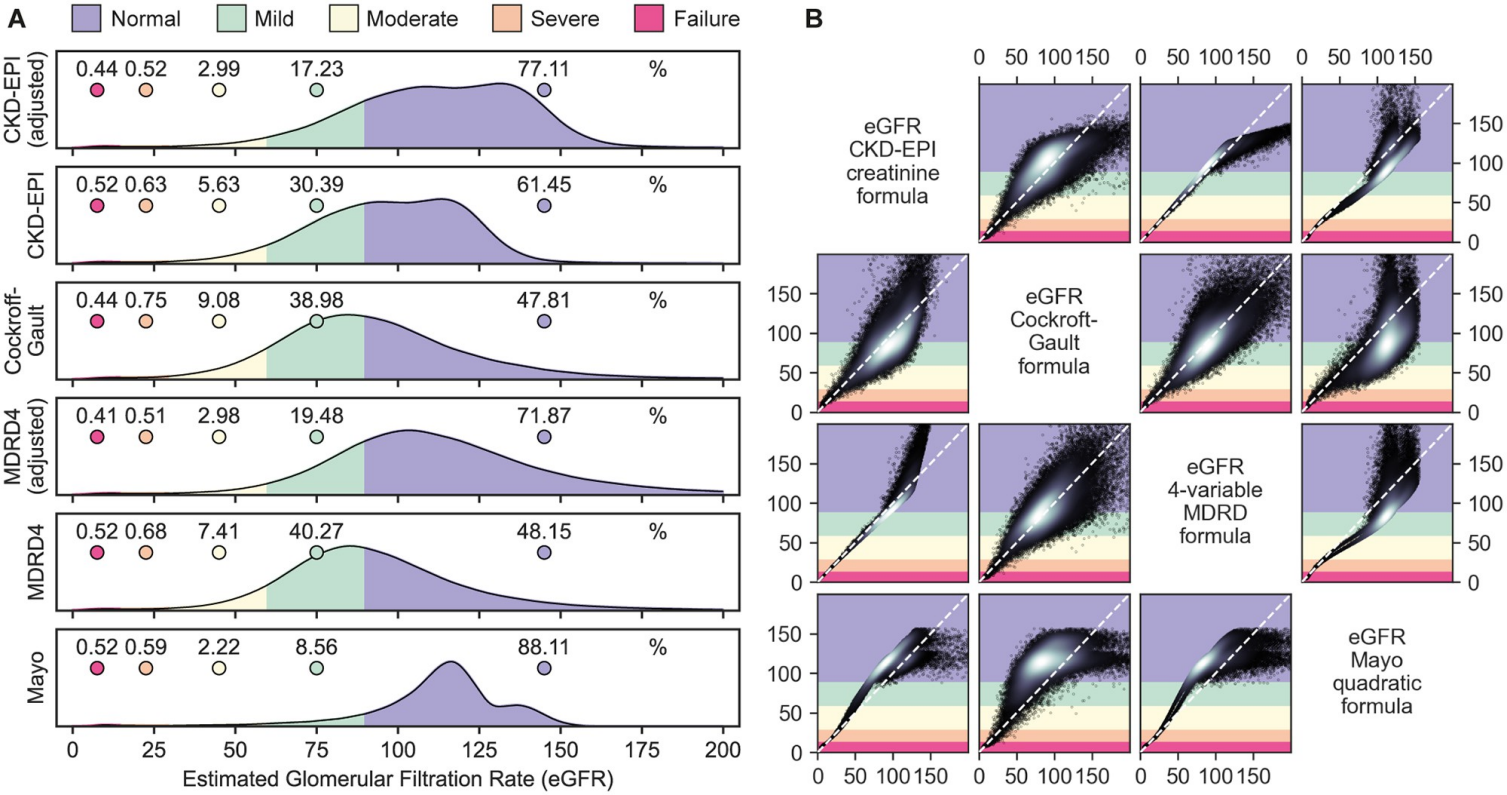
Distribution of estimated glomerular filtration rates by formula. Notes: Legend applies to Figs 4A and 4B. Category color corresponds to vertical axis in matrix. Deviation from the diagonal indicates disagreement between the two measures. White in scatter plot indicates density.

**Fig 5:**
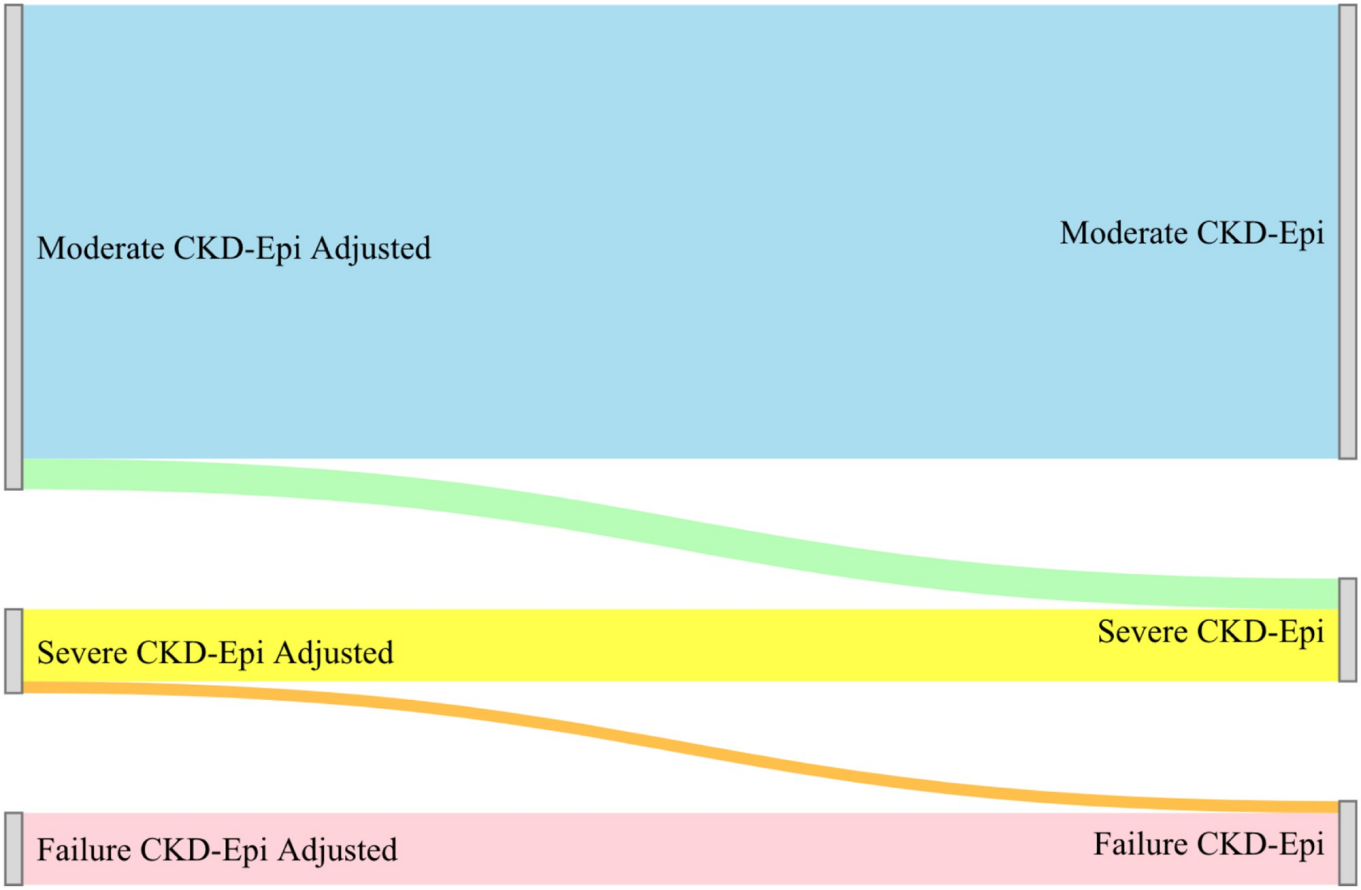
Sankey diagram unadjusted and adjusted categorization for the CKD-EPI formula.
