# Supplementary material for: Evaluation of kidney function among people living with HIV initiating antiretroviral therapy in Zambia
Source: PLOS Glob Public Health. 2022 Apr 13;2(4):e0000124. doi: 10.1371/journal.pgph.0000124 (PMC10021838; doi:10.1371/journal.pgph.0000124)
Supplement: S1 Fig — (DOCX) [file pgph.0000124.s001.docx]

**S1 Figure: Scatter Plot For eGFR (unadjusted CKD-EPI) and CD4 Cell Count (cell/mm^3^) With Linear Fit Line**
