# Supplementary material for: Evaluation of kidney function among people living with HIV initiating antiretroviral therapy in Zambia
Source: PLOS Glob Public Health. 2022 Apr 13;2(4):e0000124. doi: 10.1371/journal.pgph.0000124 (PMC10021838; doi:10.1371/journal.pgph.0000124)
Supplement: S1 Table — (DOCX) [file pgph.0000124.s002.docx]

| **S1 Table: Population Characteristics by Record of Creatinine Measure** | | | | | |
| --- | --- | --- | --- | --- | --- |
| *Factor* | *Level* | *No Creatinine Measure* | *Creatinine Measure* | *p-value* |  |
| N |  | 398681 | 68797 |  |  |
| Age | mean (SD) | 35.0 (10.0) | 35.0 (9.6) | 0.320 |  |
| Age Category | <25 years | 52513 (13.2%) | 8388 (12.2%) | <0.001 |  |
|  | 25-29 years | 72381 (18.2%) | 12610 (18.3%) |  |  |
|  | 30-34 years | 87777 (22.0%) | 15520 (22.6%) |  |  |
|  | 35-39 years | 74185 (18.6%) | 13411 (19.5%) |  |  |
|  | 40-44 years | 48912 (12.3%) | 8727 (12.7%) |  |  |
|  | 45-49 years | 29077 (7.3%) | 4746 (6.9%) |  |  |
|  | 50-54 years | 16307 (4.1%) | 2735 (4.0%) |  |  |
|  | 55+ years | 17529 (4.4%) | 2660 (3.9%) |  |  |
| Gender | Female | 254442 (63.8%) | 41138 (59.8%) | <0.001 |  |
|  | Male | 144239 (36.2%) | 27659 (40.2%) |  |  |
| Pregnant | Yes | 3366 (0.8%) | 1067 (1.6%) | <0.001 |  |
|  | No/Unknown | 395315 (99.2%) | 67730 (98.4%) |  |  |
| Diabetes Diagnosis | Yes | 1220 (0.3%) | 316 (0.5%) | <0.001 |  |
|  | No/Unknown | 197461 (99.7%) | 68481 (99.5%) |  |  |
| Body Mass Index | Underweight | 44687 (11.2%) | 14401 (20.9%) | <0.001 |  |
|  | Normal Weight | 117496 (29.5%) | 31738 (46.1%) |  |  |
|  | Overweight | 22292 (5.6%) | 5438 (7.9%) |  |  |
|  | Obese | 7704 (1.9%) | 1785 (2.6%) |  |  |
|  | Missing/Unknown | 206502 (51.8%) | 15435 (22.4%) |  |  |
| CD4 Cell Count | mean (SD) | 376.3 (263.6) | 318.8 (252.3) | <0.001 |  |
| CD4 Cell Count | >500cells/mm^3^ | 56475 (14.2%) | 8755 (12.7%) | <0.001 |  |
|  | 351-500cells/mm^3^ | 53037 (13.3%) | 11204 (16.3%) |  |  |
|  | 251-350cells/mm^3^ | 41254 (10.3%) | 10215 (14.8%) |  |  |
|  | 100-250cells/mm^3^ | 47498 (11.9%) | 14114 (20.5%) |  |  |
|  | <100cells/mm^3^ | 22852 (5.7%) | 7469 (10.9%) |  |  |
|  | Unknown | 177565 (44.5%) | 17040 (24.8%) |  |  |
| Systolic Pressure (mmHG) | mean (SD) | 113.8 (17.6) | 113.0 (17.9) | <0.001 |  |
| Diastolic Pressure (mmHG) | mean (SD) | 72.0 (13.5) | 71.5 (13.7) | <0.001 |  |
| Year of HIV Care Entry | <2011 | 125604 (31.5%) | 3939 (5.7%) | <0.001 |  |
|  | 2011 | 34554 (8.7%) | 12753 (18.5%) |  |  |
|  | 2012 | 31268 (7.8%) | 12506 (18.2%) |  |  |
|  | 2013 | 32293 (8.1%) | 12831 (18.7%) |  |  |
|  | 2014 | 36121 (9.1%) | 11676 (17.0%) |  |  |
|  | 2015 | 40736 (10.2%) | 8006 (11.6%) |  |  |
|  | 2016 | 41445 (10.4%) | 6144 (8.9%) |  |  |
|  | 2017 | 56660 (14.2%) | 942 (1.4%) |  |  |

Note: p-values for categorical variables are based on Chi-squared test and continuous variables based on t-test
