# Supplementary material for: Evaluation of kidney function among people living with HIV initiating antiretroviral therapy in Zambia
Source: PLOS Glob Public Health. 2022 Apr 13;2(4):e0000124. doi: 10.1371/journal.pgph.0000124 (PMC10021838; doi:10.1371/journal.pgph.0000124)
Supplement: S2 Table — (DOCX) [file pgph.0000124.s003.docx]

| **S2 Table: Population Characteristics by Record of Multiple Creatinine Measures** | | | | | |
| --- | --- | --- | --- | --- | --- |
| *Factor* | *Level* | *Single Measure* | *Multiple Measures* | *p-value* |  |
| N |  | 65252 | 3282 |  |  |
| Sex | Female | 38525 (59.0%) | 2414 (73.6%) | <0.001 |  |
|  | Male | 26727 (41.0%) | 868 (26.4%) |  |  |
| Age Category | <25 years | 8168 (12.5%) | 470 (14.3%) | <0.001 |  |
|  | 25-29 years | 12170 (18.7%) | 693 (21.1%) |  |  |
|  | 30-34 years | 14746 (22.6%) | 770 (23.5%) |  |  |
|  | 35-39 years | 12696 (19.3%) | 582 (17.7%) |  |  |
|  | 40-44 years | 8129 (12.5%) | 362 (11.0%) |  |  |
|  | 45-49 years | 4425 (6.8%) | 186 (5.7%) |  |  |
|  | 50-54 years | 2539 (3.9%) | 128 (3.9) |  |  |
|  | 55+ | 2469 (3.8%) | 91 (2.7%) |  |  |
| Body Mass Index | Under Weight | 2542 (3.9%) | 19 (0.6%) | <0.001 |  |
|  | Normal Weight | 6021 (9.2%) | 72 (2.2%) |  |  |
|  | Overweight | 1240 (1.9%) | 17 (0.5%) |  |  |
|  | Obese | 454 (0.7%) | 5 (0.2%) |  |  |
|  | Missing/Unknown | 54995 (84.3%) | 3169 (96.6%) |  |  |
| Diabetes | Yes | 65 (0.1%) | 1 (<1%) | 0.378 |  |
|  | No/Unknown | 65187 (99.9%) | 3281 (100%) |  |  |
| Blood Pressure Category | Hypotensive | 3675 (5.6%) | 62 (1.9%) | <0.001 |  |
|  | Normotensive | 19973 (30.6%) | 692 (21.1%) |  |  |
|  | Pre-Hypertensive | 3561 (5.5%) | 116 (3.5%) |  |  |
|  | Hypertensive Stage I | 6917 (10/6%) | 262 (8.0%) |  |  |
|  | Hypertensive Stage II | 3302 (5.1%) | 160 (4.9%) |  |  |
|  | Severe Hypertension | 799 (1.2%) | 49 (1.5%) |  |  |
|  | Missing/Unknown | 27025 (41.4%) | 1941 (59.1%) |  |  |
| CD4 Cell Count | >500cells/mm^3^ | 7896 (12.1%) | 1187 (25.3%) | <0.001 |  |
|  | 351-500cells/mm^3^ | 10143 (15.5%) | 1028 (31.3%) |  |  |
|  | 251-350cells/mm^3^ | 9683 (14.8%) | 498 (15.2%) |  |  |
|  | 100-250cells/mm^3^ | 13771 (21.1%) | 291 (8.9%) |  |  |
|  | <100cells/mm^3^ | 7311 (11.2%) | 142 (4.3%) |  |  |
|  | Missing/Unknown | 16448 (10.0%) | 494 (15.1%) |  |  |

Note: Factors, sex, age category, body mass index, blood pressure category, and CD4 cell count p-values based of Chi-squared test; factor diabetes based on Fisher’s Exact Test
