# Supplementary material for: Evaluation of kidney function among people living with HIV initiating antiretroviral therapy in Zambia
Source: PLOS Glob Public Health. 2022 Apr 13;2(4):e0000124. doi: 10.1371/journal.pgph.0000124 (PMC10021838; doi:10.1371/journal.pgph.0000124)
Supplement: S3 Table — (DOCX) [file pgph.0000124.s004.docx]

**S3 Table: Crude and Adjusted Prevalence Ratios (PR) for Limited eGFR (<60ml/min/1.73m2)**

| *Covariate* | *Level* | *Crude* | | *Adjusted* | |
| --- | --- | --- | --- | --- | --- |
|  |  | *PR* | *95% CI* | *PR* | *95% CI* |
| Sex | Female | ref | ref | ref | ref |
|  | Male | 0.95 | (0.90, 1.00) | 0.75 | (0.70, 0.79) |
| Age Category | 17-24 years | ref | ref | ref | ref |
|  | 25-29 years | 1.36 | (1.17, 1.57) | 1.37 | (1.16, 1.62) |
|  | 30-34 years | 1.71 | (1.48, 1.96) | 1.73 | (1.48, 2.012 |
|  | 35-39 years | 2.37 | (2.07, 2.72) | 2.41 | (2.04, 2.85) |
|  | 40-44 years | 3.20 | (2.79, 3.69) | 3.20 | (2.62, 3.90) |
|  | 45-49 years | 4.33 | (3.75, 5.01) | 4.23 | (3.39, 5.29) |
|  | 50-54 years | 5.83 | (5.02, 6.77) | 5.60 | (4.40, 7.13) |
|  | 55-59 years | 7.64 | (6.51, 8.95) | 7.20 | (5.62, 9.21) |
|  | 60-64 years | 9.54 | (8.00, 11.37) | 9.10 | (7.14, 11.60) |
|  | 65+ years | 11.22 | (9.40, 13.40) | 10.39 | (7.88, 13.70) |
| Body Mass Index | Under Weight | ref | ref | ref | ref |
|  | Normal Weight | 0.74 | (0.63, 0.87) | 0.85 | (0.71, 1.01) |
|  | Overweight | 0.84 | (0.66, 1.06) | 0.91 | (0.67, 1.23) |
|  | Obese | 1.00 | (0.72, 1.38) | 1.07 | (0.83, 1.37) |
|  | Unknown | 0.78 | (0.69, 0.89) | 1.01 | (0.86, 1.19) |
| Blood Pressure Category | Hypotensive | 1.43 | (1.27, 1.60) | 1.35 | (1.19, 1.53) |
|  | Normotensive | ref | ref | ref | ref |
|  | Pre-Hypertensive | 0.94 | (0.82, 1.07) | 0.91 | (0.80, 1.03) |
|  | Hypertensive Stage I | 1.11 | (1.00, 1.22) | 1.03 | (0.93, 1.13) |
|  | Hypertensive Stage II | 1.57 | (1.42, 1.74) | 1.23 | (1.11, 1.36) |
|  | Severe Hypertension | 2.81 | (2.22, 3.54) | 1.64 | (1.35, 1.98) |
|  | Unknown | 0.97 | (0.91, 1.04) | 1.03 | (0.94, 1.13) |
| CD4 Cell Count | >500 | ref | ref | ref | ref |
|  | 351-500 | 1.00 | (0.89, 1.13) | 0.94 | (0.84, 1.05) |
|  | 251-350 | 1.24 | (1.10, 1.40) | 1.14 | (0.99, 1.30) |
|  | 100-250 | 1.62 | (1.45, 1.77) | 1.39 | (1.24, 1.56) |
|  | <100 | 2.10 | (1.88, 2.35) | 1.79 | (1.53, 2.09) |
|  | Unknown | 1.60 | (1.44, 1.77) | 1.46 | (1.29, 1.66) |

Note: PR – prevalence ratio, CI – confidence interval, crude and adjusted analysis allow random effect at the facility level, adjusted analysis adjustment for sex, age, BMI, blood pressure category and CD4 cell count
