# Supplementary material for: Evaluation of kidney function among people living with HIV initiating antiretroviral therapy in Zambia
Source: PLOS Glob Public Health. 2022 Apr 13;2(4):e0000124. doi: 10.1371/journal.pgph.0000124 (PMC10021838; doi:10.1371/journal.pgph.0000124)
Supplement: Attachment — Submitted filename: review_response-22nov2021.pdf [file pgph.0000124.s005.pdf]

Centre for Infectious Disease Research Zambia
Implementation Science Unit

Jake M. Pry, PhD, MPH
Research Scientist – Epidemiologist
jake.pry@cidrz.org | +260 96 3088750 | +1-936-661-6885

Siyan Yi, MD, MHSc, PhD
Academic Editor
PLOS Global Public Health

Dear Dr. Yi,

Many thanks for reviewing our manuscript (PGPH-D-21-00415) titled "Evaluation of kidney function among people living with HIV initiating antiretroviral therapy in Zambia".

On behalf of my colleagues, I wanted to thank you and the reviewers for your thoughtful review and feedback. We have reviewed each comment carefully and have made the changes noted below, which we believe have substantially strengthened the manuscript. We have enclosed two versions of the revised manuscript in Word (.docx)—one with changes highlighted under "track changes" and a second "clean" version with all changes accepted.

Please find here our point-by-point responses to reviewer comments, organized by headings taken from your March 20, 2020 email (also, please note that for ease of reference the lines below refer to the "clean" version of the revised manuscript):

Journal Requirements:

1. Please amend your detailed Financial Disclosure statement. This is published with the article, therefore should be completed in full sentences and contain the exact wording you wish to be published.

i). Please include all sources of funding (financial or material support) for your study. List the grants (with grant number) or organizations (with url) that supported your study, including funding received from your institution.
ii). State the initials, alongside each funding source, of each author to receive each grant.
iii). State what role the funders took in the study. If the funders had no role in your study, please state: "The funders had no role in study design, data collection and analysis, decision to publish, or preparation of the manuscript."

iv). If any authors received a salary from any of your funders, please state which authors and which funders.

Response: This has been included as requested. The funding statement is as follows, "Support for this work is provided by the President's Emergency Plan for AIDS Relief (PEPFAR) and Centers for

Infectious Disease Research Zambia through under study protocol approved by the U.S. Centers for Disease Control & Prevention (2018-381), University of Zambia Biomedical Research Ethics Committee (011-12- 17), University of North Carolina at Chapel Hill, USA (18- 0854) and the Institutional Review Board at Washington University, St. Louis, USA (2019-11143) without requiring patient consent for review of de-identified, routinely collected data."

2. Please ensure that the funders and grant numbers match between the Financial Disclosure field and the Funding Information tab in your submission form. Note that the funders must be provided in the same order in both places as well.

Response: We have reviewed this carefully to ensure that all funding information matches in the Financial Disclosure field and the Funding Information tab.

3. Please update the completed 'Competing Interests' statement, including any COIs declared by your co-authors. If you have no competing interests to declare, please state "The authors have declared that no competing interests exist". Otherwise please declare all competing interests beginning with the statement "I have read the journal's policy and the authors of this manuscript have the following competing interests:"

Response: We have updated 'Competing Interests' statement accordingly. It reads, "There are not competing interests to report"

4. In the online submission form, you indicated that "De-identified data for this work will be made available upon request.". All PLOS journals now require all data underlying the findings described in their manuscript to be freely available to other researchers, either 1. In a public repository, 2. Within the manuscript itself, or 3. Uploaded as supplementary information. This policy applies to all data except where public deposition would breach compliance with the protocol approved by your research ethics board. If your data cannot be made publicly available for ethical or legal reasons (e.g., public availability would compromise patient privacy), please explain your reasons by return email and your exemption request will be escalated to the editor for approval. Your exemption request will be handled independently and will not hold up the peer review process, but will need to be resolved should your manuscript be accepted for publication. One of the Editorial team will then be in touch if there are any issues.

Response: Not able to make publcially available. The Government of Zambia allows data sharing after a review of data queries ensures the appropriateness of its intended use. To request data access, contact the CIDRZ Ethics and Compliance Committee Chair/Chief Scientific Officer, Dr. Roma Chilengi, Roma.chilengi@cidrz.org, or the Secretary to the Committee/Head of Research Operations, Ms. Hope Mwanyungwi, Hope.Mwanyungwi@cidrz.org, mentioning the intended use for the data.

5. Please provide separate figure files in .tif or .eps format only and remove any figures embedded in your manuscript file. Please ensure that all files are under our size limit of 20MB.

For more information about how to convert your figure files please see our guidelines: https://journals.plos.org/globalpublichealth/s/figures

Once you've converted your files to .tif or .eps, please also make sure that your figures meet our format requirements.

Response: We have uploaded figures in the format requested.

6. Tables should not be uploaded as individual files. Please remove these files and include the tables in your manuscript file.

Response: We have removed the copy of Table 1 as an attachment and it is present in the manuscript file only.

7. We notice that your supplementary figures are included in the manuscript file. Please remove them and upload them with the file type 'Supporting Information'. Please ensure that all Supporting Information files are included correctly and that each one has a legend listed in the manuscript after the references list.

Response: We have removed all supplementary figures from the manuscript and re-uploaded them as Supporting Information in accordance with instructions.

8. Please ensure that you refer to Figure 5 in your text as, if accepted, production will need this reference to link the reader to the figure.

Response: We have now included a reference to Figure 5 in the revised results section.

9. Please match your Figures' Descriptions with their corresponding file names by renaming them accordingly.

Response: We have renamed file names in the revised manuscript to match figures' descriptions.

Additional Editor Comments (if provided):

[Note: HTML markup is below. Please do not edit.] Reviewers' comments:
Reviewer's Responses to Questions

Begin response to specific reviewer feedback:

Reviewer #1: The authors are to be congratulated on their manuscript entitled 'Evaluation of kidney function among people living with HIV initiating antiretroviral therapy in Zambia' which utilises a large national data set to address an important issue of renal disease in people living with HIV in Zambia. These data will add significantly to existing knowledge of the burden of renal impairment in PLWH and its risk factors and will contribute towards an emerging collection of much needed data for national and international non-communicable disease management policies.

There are some aspects which I think, if addressed, could improve the strength of the manuscript.

Response: Thank you so much for your thoughtful review and valuable feedback. We have addressed the reviewer's points one-by-one below.

Major comments
Overall
1. Readers may benefit from a little more information on CIDRZ sites (perhaps there is a reference

which can be included for this?). What is particularly important is giving an idea of the types of settings these data cover. Are they general outpatients, specific HIV clinics, inpatient settings, acute emergency care etc?

Response: Thank you for this comment. We have added language in the methods section to include the scope of the Centre for Infectious Disease Research Zambia and additional detail regarding the clinic settings included in these analyses. Please see lines 105-113.

2. Further to this, it is critical that the reader has a clear understanding of who the included patients are from a clinical point of view. Is each data point one patient initiating ART (as indicated in the title)? Or are the data points from different stages across the HIV disease journey (as inferred in the abstract – "among a cohort of PLHIV with an HIV care visit" and the methods section – "All individuals with an HIV care clinic visit")? If these are all data points from ART initiation and some are in an acute inpatient setting whilst others are in an outpatient clinic setting, it will be important to note that there may be significant differences within the cohort in terms of clinical disease. For example, those who have their HIV diagnosed as an inpatient may be much more clinically unwell, with sepsis for example, which may affect renal function.

Response: Thank you for pointing this out. We have revised the language to explain that measures are primarily among those initiating ART; however, there are some repeat measures recorded in the dataset. What we hope this analysis demonstrates is that it is routine for individuals to have a serum creatinine at ART initiation, but that follow-up measures may be done at the discretion of local clinicians. Please see lines 121-123.

3. The manuscript would benefit from standardisation of the terms used to define the outcome of renal impairment. These change throughout the manuscript which can make it difficult to follow (terms used include: eGFR measures, kidney impairment, moderate kidney function impairment, kidney function impairment, at least moderately impaired kidney function, at least moderately to severely impaired kidney function). Further, it is very important to differentiate between acute kidney injury and chronic kidney disease. It will be difficult to do this with data from one time point but this should be discussed, and implications addressed.

Response: We agree with this important point – thank you. We have resolved the variety of terms used to imply kidney impairment and standardized the term to kidney function impairment throughout the revised manuscript. It is not possible to to distiguish between chronic kidney disease and acute kidney injury with the current analysis and we have specified this in the limitations in the discussion section. "Additionally, as we are not able to parse chronic kidney impairment from acute kidney injury (AKI) it is possible that some of those with decreased eGFR measures could be experiencing acute kidney injury and not necessarily indicative of chronic kidney disease." Lines 511-514

4. It would be nice to have an explanation of why the reported formulae were chosen. In particular, I would advise the authors to re-consider the inclusion of measures that adjust for race. There has been much international criticism of the inclusion of race in renal function calculations, arguing that race is a social construct rather than a biological one and including it in biological calculations disadvantages Black African populations.

Response: We attempted to present the gamut of estimated glomerular filtration rate caluculations with the intent to highlight the differences in kidney function impairment category proportions. Main effects are assessed using unadjusted CKD-EPI measures. We have included additional

discussion around race adjustement in the discussion section “We calculated both adjusted and unadjusted eGFR using the CKD-Epi and MDRD-4 equations. The race adjusted proportion significantly lower of individuals in the normal, mild and moderate categories compared to the not race adjusted for both the CKD-Epi and the MDRD-4 formulae. The proportion of those in the severe and kidney failure categories (eGFR < 30 ml/min/1.73m2 for the race adjusted and not race adjusted CKD-Epi and MDRD-4 equations did not differ significantly. This could have implications for programs seeking to address kidney function impairment depending on the eGFR threshold for referral or renal care.

“

Background
1. A little more detailed discussion of current knowledge of incidence and prevalence of AKI and CKD in PLWH in SSA would be beneficial

Response: Thank you for this point. We have added language to the background section of the revised manuscript regarding what is known about the prevalence of AKI in sub-Saharan Africa. A large prospective cohort estimated annual AKI incidence of 3.4% among those admitted to hospital in Cape Town South Africa (Dlamini et al, PLoS One 2017). Chronic kidney disease was estimated to be 10.0% in 2017 in Zambia (GDB Chronic Kidney Disease Collaboration, The Lancet 2020)

2. It might be useful to understand whether there are guidelines within the CIDRZ facilities for measurement of blood pressure, weight/height and diabetes, or whether these are carried out on an ad hoc basis when there is a clinical concern.

Response: Measures of height and weight are most often captured at the initial history and physical examination for persons initiating ART; however, collection of these measures may depend on availability/functionality of equipment at the clinic as well as the clinic burden at the time of intake. There are no guidelines restricting the timing for the collection of height and/or weight to those that may be considered for any reason at a perceived different risk for non-communicable diseases. To make this point clearer, we have added the following description to the revised methods section, “Though the framework for capturing covariates exists in the electronic HIV medical record it is important to note that they are not required by the system.“

“

Methods
1. line 114. Why was there a modification to the AHA/ACC hypertension guidelines definition of severe hypertension?

Response: We thank the reviewer for seeking clarification of this point. We felt that a single patient group including such a wide range of systolic pressures (e.g., 140mmHg and 190mmHg) should be more completely differentiated to better inform meaningful insights. Additional thresholds within this category were determined from previous literature, additional citations were included. Line 187

Results
1. Overall, it is a little difficult to grasp the main findings of the study; this section might benefit from a little more focussing of the important messages

Response: We thank the reviewer for this point and agree that the key findings were not as clear as they could have been. In response, we have restructured the results section to improve the flow and wit them aim to more clearly present results.

2. Line 214 “Obesity was also correlated....”. Please quote the numbers in the text to make this sentence more specific for the reader.

Response: Thank you for this point. However, this statement was omitted in the revised manuscript to improve clarity.

3. Line 215. “There was a substantial amount...”. Please report exactly how much data was missing (it is the vast majority of included participants).

Response: We have added the exact measure of missing data in the revised results section (84.9%).

4. Line 217. “.. the data available show a slightly higher proportion of...” It is unclear what is being compared here. Higher proportion than what?

Response: Thank you, we have omitted inferences on body mass index given the scant data available.

5. Line 217. “Crude prevalence ratio...are very similar”. Again, unclear what is being compared here. Very similar to what? Also need to include the numbers in this sentence.

Response: For clarity we have omitted this sentence and focused on better presenting key results.

6. Overall, I would have reservations about reporting the obesity data at all given that it is in such a small proportion of the cohort and is likely to be subject to considerable bias (eg only being measured in those who are clinically unwell).

Response: Thank you for this point. We have omitted body mass index in Table two and added the original table two as a supplementary table (Table S3).

7. line 236. “There were 3,216 with multiple creatinine measures”. Please clarify 3,216 what. I presume PLWH included in the renal analysis cohort. Although this doesn’t seem to tie in with the earlier assertion that 3209 had multiple measurements?

Response: We have amended this sentence for clarity: “There were 3,209 individuals in the analysis set with multiple creatinine measures spaced by a median of 210 days (IQR: 133-383 days).”

8. Line 238: “Among those with at least...”. This sentence doesn’t make sense to me. I wonder if it would perhaps help to omit the part that says “Among those with at least two creatinine measures” as it seems to me that this analysis would have been done within the bigger cohort?

Response: We have omitted this sentence to improve readability and clarity as suggested by the reviewer.

9. Line 241: “Among those with multiple eGFR measures at 3-12 months...”. It would be beneficial to have specific numbers here to support the observation. This finding would also make me concerned that (if the overall cohort was indeed from ART initiation timepoint), a lot of what has been observed is due to patients being clinically unwell at ART initiation, which then resolves with treatment and/or ART. This would lead to quite a different overall conclusion for the article if it were the case.

Response: We thank the reviewer for this important point. We have further clarified this to indicate that measures tend to decrease slightly at follow-up and added a supplemental table to describe the characteristics of those with multiple measures compared to those with a single measure.

10. Line 247: It might be helpful to include a few words here on what the adjusted models are comparing

Response: We have added the following sentence for improved context, “We calculated mixed effects Poisson regression estimates for eGFR <60ml/min (CKD-EPI) adjusted for age, sex, body mass index, blood pressure category, and CD4 cell count allowing for random effects at the facility level.”

11. 248 -258: It's not clear from how this paragraph is phrased that it is reporting the results of an adjusted model. The first sentence could be along the lines of 'a logistic regression model examining cross sectional risk factors for moderate or severe renal impairment was constructed'. Independent risk factors included x,y and z (can list in order of association and give their odds ratios and confidence intervals after each).

Response: We have included an introduction sentence to better orient the reader to the adjusted model findings at the beginning of the adjusted analysis sub-heading ” We calculated mixed effects Poisson regression estimates for eGFR <60ml/min (CKD-EPI) adjusted for age, sex, body mass index, blood pressure category, and CD4 cell count allowing for random effects at the facility level.”

Discussion

1. Line 282: “at least moderately to severely impaired kidney function” isn’t quite clear and doesn’t fit with definitions used in rest of text.

Response: Thank you for this comment. We have revised the language to refer to eGFR range rather than “moderate” or “severe”.

2. Line 296: “Our estimates for kidney function are also higher”. It is unclear what this sentence means.

Response: Thank you for pointing this out. We have revised this to improve the clarity, “The median eGFR (per Cockcroft-Gault formula) of 90.5 ml/min/1.73m2 (IQR: 73.7, 110.9) is 5.5ml/min/1.73m2 lower than that reported by Mocroft et al at 96ml/min/1.73m2 (IQR: 82, 111)”

3. Line 308: “chronic kidney disease as we show here”. I would have concerns about this. It is not clear to me that what is being presented in this paper is an assessment of chronic kidney disease (see comments above). This needs to be clarified.

Centre for Infectious Disease Research in Zambia
Plot # 34620 Corner of Lukasu & Danny Pule Roads, Mass Media, P.O. Box 34681 Lusaka, Zambia 10101
Reception: +260 211 242 257 - 63
Email: info@cidrz.org @cidrzinfo https://www.facebook.com/CIDRZ www.cidrz.org

Response: Thank you for identifying this point of confusion. We have revised narrative to improve clarity "These data serve as evidence that routine data may help jumpstart an understanding of the burden of kidney function impairment as well as guide the response to underlying non-communicable co-morbidities like kidney function impairment and high blood pressure."

Line 348: "We do not suspect differential bias to be associated with data missingness". I would have concerns that there might indeed be bias with the risk factor data. Please provide information that would reassure the reader that these data are not biased (as per comment above on explaining local guidelines on weight measurement).

Response: Thank you for this important consideration. We have included a supplementary table comparing the larger population to those with classifiable eGFR measures (Table S1). Focusing on CD4 cell count, likely the best indicator of HIV progression, there is a difference in those with CD4 <100cells/mm^3 however because a larger percentage of observations among those with a creatinine measures initiated care from 2011-2016, an era with CD4 cell count was a criteria for ART intiaition this slight tenancy toward lower CD4 cell counts is expected.

Line 349: "Another limitation is the ....". This sentence is not clear to me. Is the measurement performed as standard of care, or is it routinely collected as clinical cause/judgement? In particular, is it not likely that those with repeated measurements are subject to clinical judgement?

Response: Thank you – we have revised this statement to improve clarity, "Another limitation is limited medical notes and facility-level clinical context available to understand why some individuals had multiple creatinine measures in the medical record." We compared this subset of individuals in supplemental table 2 and found that those with multiple measures are slightly younger and have a higher CD4 cell count compared to those with baseline only measures.

Line 360: Again, point as above, I'm not sure the data presented in this paper has provided evidence on "those engaged in HIV care at increased risk for chronic kidney disease".

Response: Thank you for this point. We have refined the language as follows, "Differentiated service delivery models could be a promising model to reinforce referral and kidney function monitoring among those initiating ART with kidney function impairment (eGFR <60 ml/min/1.73m2)."

Tables

Table 1

1. Looking at these data, I wonder what the p values are telling us practically. There are a lot of groups being compared. As a minimum, please insert a footnote with information on what statistical test was used and what it compared.

Response: We have added a footnote detailing the type of test associated with the p-value indicated.

2. Conversely, there is an extremely high proportion of missingness for weight and diabetes categories and I wonder whether tests of statistical comparison are appropriate here?

Response: We appreciarte the reviewer's point, however, we think it is beneficial to have some objective measure comparing the groups.

3. I find it interesting that approximately half of patients in severe or failure categories are on TDF. This is despite the authors' explanation that renal function is tested at ART initiation to help decide whether TDF can be given safely. I wonder whether this practice has changed throughout the course of the study. It might be worth a line of explanation on this.

Response: We agree with thre reivewer's point. We have revised the discussion section to note the possiblility that those with advanced HIV should be started on the available regimen immediately rather than waiting. "We observed parallel decreasing in the proportion of individuals receiving a TDF contain regimen and eGFR category until eGFR<155ml/min/1.73m2. It is possible that some individuals with low eGFR measures presented to the clinic as more ill and were initiated on a TDF-containing first line to avoid any delay in ART initiation and later switched to a non-TDF-containing regimen."

Table 2
1. I find the linear correlation between CD4 T cell count and risk of renal impairment interesting. This could of course be related to acute kidney injury from intercurrent illness, but it might be worth highlighting this in the text rather than, or in addition to, comparing low category with high.

Response: Thank you. We have added a scatter plot illustrating this relationship as supplemental figure one.

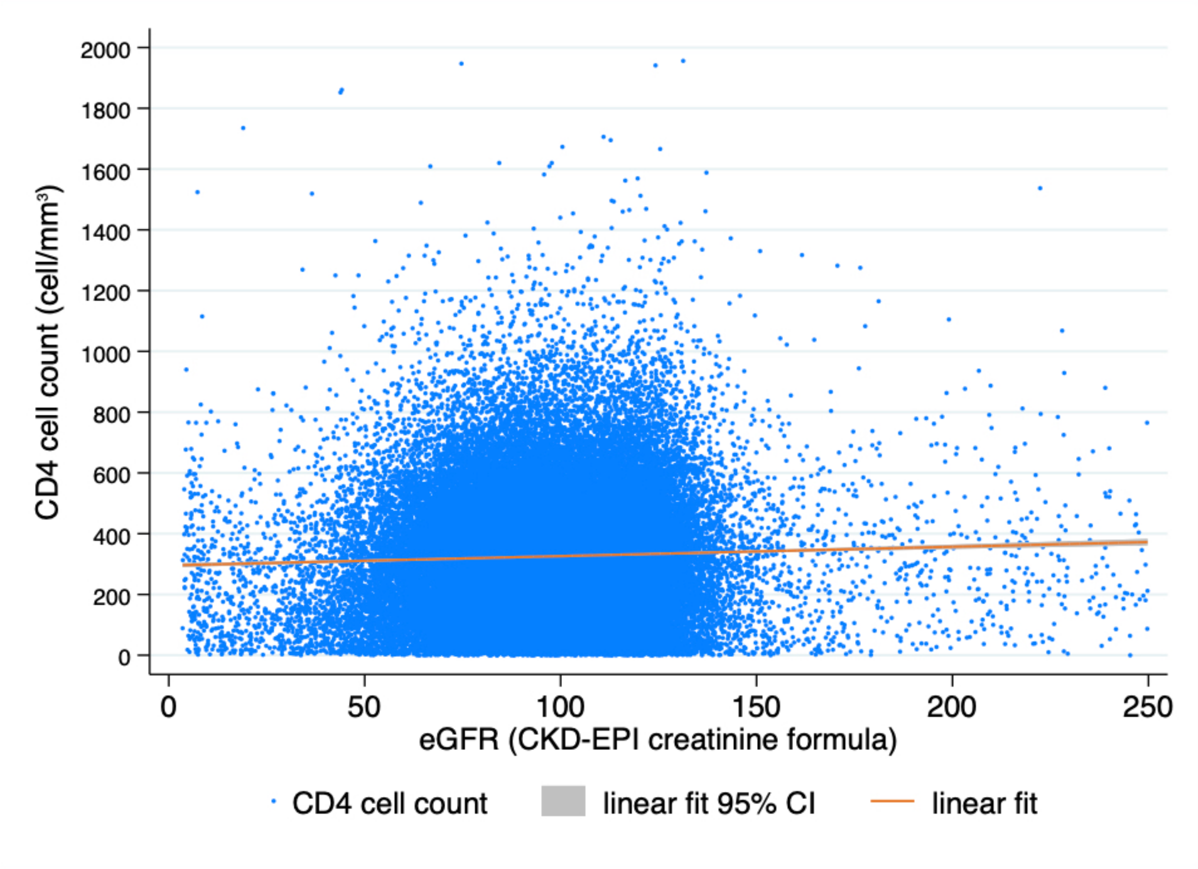

2. It would be beneficial to have a small footnote explaining what the analyses were adjusted for and how the models were constructed. This does not seem to be detailed in the methods section.

Response: We have added a footnote and narrative in the methods section describing the adjustment considerations.

Minor comments

Abstract

Methods section should read “across seven of the ten” (instead of or)

Response: This has been amended as requested

Background

Line 89: ‘predictors of kidney impairment’. Can the authors please clarify that they assessed predictors in a longitudinal analysis, rather than cross sectional. If this is cross sectional analysis, can they please change to risk factors or associations? Methods

Response: Thank you for this note. We have added a “design” heading at the beginning of the methods section to better contextualize the cross-sectional from the longitudinal.

line 126: “National Kidney Score”. Please indicate which nation this refers to.

Response: We have corrected the reference to read “United States National Kidney Foundation”

Line 152: Please change to “Multiple imputation was considered where missingness was <30%”

Response: changed per reviewer suggestion

Line 153: Please change to “Categories for BMI are defined according to World Health Organization criteria”.

Response: changed per reviewer suggestion

Line 160: I’m not sure a description of what graphs were made is needed if you need to save words.

Response: we have omitted per reviewer suggestion

Results

Line 186: Please change to Prevalence of Kidney Function Impairment (or alternative standardised outcome term)

Response: changed per reviewer suggestion to “Prevalence of Kidney Function Impairment”

Line 190: “Severe impairment and kidney failure”. Perhaps “Severe impairment or kidney failure” might be clearer? Line 225: Please specify if the protease inhibitor regime is also only first line as for TDF and non-TDF

Response: changed per reviewer suggestion to “Severe kidney function impairment or kidney failure…”

Line 227: This sentence might read better if medians, IQR and p value were inserted together at the end.

Response: We have omitted IQR measures to improve clarity "Individuals prescribed a non-TDF-containing ART regimen had a significantly lower eGFR with a median of 78.4 compared to those on a TDF-containing ART regimen with a median of 99.8 (Pearson p-value: <0.001)."

Line 228: "Additionally, we illustrate....". This sentence is unclear, I'm not sure what is being said here. Please rephrase.

Response: we have amended to "Additionally, we illustrate the decreasing trend in proportion of individuals receiving a TDF-containing ART regimen with decreasing eGFR, with the exception of those with the lowest eGFR category (<15 ml/min/1.73m2) (Figure 2)." to improve clarity

Discussion

Line 285: I'm not sure what is referred to by "screen for TDF tolerance". Perhaps "TDF suitability" might be a clearer term?

Response: we have amended to "TDF" suitability for clarity

Line 296: There is a missing bracket at the end of the CI figures.

Response: thank you, we have amended

Line 308: I would delete the word "other" from this sentence as HIV is not a non-communicable disease

Response: we have revised and omitted "other"

Line 318: please change effect to affect

Response: we have corrected effect to affect

Line 322: "As DTG is now a WHO recommended..." This sentence seems incomplete?

Response: we have moved and incorporated this sentence to read "As Dolutegravir continues to be rolled out, it will be increasingly important to monitor potential DTG regimen associated risks for increased body mass index, as well as account for the more direct creatinine clearance effects among those receiving a DTG containing ART regimen."

Line 329: change contain to containing

Response: we have corrected per suggestion

Line 332: not sure what is meant by "care referral and incorporation"?

Response: we have amended sentence to improve clarity "There is a non-trivial amount of missing data in the EMR which may limit its utility as a tool for renal care referral and increased incorporation of routine measures into the Zambia HIV care guidelines."

Line 332: the word "is" is repeated

Response: we have amended to read "Zambia is in the process…"

Line 340: "A critical challenge...". Is this sentence complete? Refer for what?

Reseponse: we have amened to read "Critical challenges to integrating NCD care at HIV care sites including limited clinic space, over-crowding, and availability of clinical staff to conduct screening and referral might be addressed through the continued uptake and expansion of differentiated HIV service delivery"

Line 340: "It is possible with..." It isn't clear what this sentence is trying to say.

Response: we have amended to "It might be possible and important to design a differentiated service delivery model for those initiating HIV care with kidney function impairment…"

Line 348: "fieldsin" requires a space

Response: we have amended to "fields in"

Line 351: this sentence is disjointed, please rephrase for clarity.

Response: we have amended to "Another limitation is limited context available to understand why some individuals had multiple creatinine measures in the medical record."

Acknowledgements
Line 365: You may wish to change received to receive.

Response: thank you, we have corrected to "receive"

Tables
Table 1: Please indicate whether they are all first line regimes or not.

Response: we have amended to "First Line TDF-Containing"

Reviewer #2: 1. What is this manuscript all about?

In this study, they set out to determine the prevalence of kidney dysfunction/kidney failure defined as having moderate kidney dysfunction eGFR<60mL/min (moderate kidney function impairment) in unspecified/different censoring time within years. compare the different criteria for determining estimated glomerular filtration rate and model the predictors of the trajectory of kidney function following initiating of ART based therapy. They had a sample size of 68 534 with 72933 observations. They included anyone with at least baseline creatinine measurement and used mixed effect Poisson model to model moderate dysfunction which was defined as eGFR < 60ml/min/1.73m2.

General comment:
The research needs to be well focused with clearly outlined objectives to achieve. The analysis done and chose of statistical methods do not seem to meet the question they intended to answer and the conclusion made were not supported by data; this was true about the discussion as well. They had a lot of missing data and trivialized that fact in an interest to have a very large sample size. There is more analysis, review and probably effective methodological amendments they need to do to make the work clearer and publishable.

2. Have the authors identified the question and key claims and context in the introduction? NO, this has not been well done. The research did not have a well-focused question to answer and seemed to be nebulous

Response, we have amended the background second language to more clearly outline the hypothesis being tested as follows "Better estimates of kidney function impairment can provide evidence and motivating rationale to expand NCD care guidance at ART care facilities and for HIV/NCD care integration to improve outcomes among those in HIV care in Zambia."

3. Have they discussed related research? How does the study fit in the related research? They have referenced some research but they have not tied in their study well and do not clearly demonstrate how theirs adds knew knowledge or innovation.

Response: we have framed the work in this way "We calculated eGFR from routine, programmatic HIV care measures recorded in the national electronic HIV medical record. Leveraging information in the medical record we assessed predictors of kidney function impairment (<60ml/min/1.73m2) using regression analysis. We also evaluated correlation between TDF-containing regimens and eGFR and compared six different formulae for eGFR. These findings can guide policy, care recommendations, and represent the opportunity for kidney/HIV care integration to improve health outcomes through spotlighting this high-risk group at the national level"

4. Do the figures and tables make sense given the results? The tables may be combined for clarity. They also need a key for statistical methods used for the test of the null hypotheses and are better placed right below the results.

Response: we have added details on the significance test applied in the footnote of table 1 "Note: p-value for continuous variables were calculated with t-test and p-values for categorical variables were calculated with Chi-squared test, ART-antiretroviral therapy, IQR-interquartile range"

"

5. Methods and study design. Do the methods make sense and follow appropriate reporting guidelines? The study design they mentioned was cross-sectional but it appears this was supposed to be a retrospective cohort study. They followed patients that for onset of moderate dysfunction after therapy.

Response: We have amended the methods section to include a design subheading, „Design
We conducted a cross-sectional analysis on estimated glomerular filtration rate among individuals initiating ART in Zambia using the national electronic HIV medical record. Additionally, among those with multiple measures in the HIV medical record we conducted descriptive analysis to identify trends in eGFR during a two years of follow-up period

6. Are the conclusions supported by the data and results?
NO, a lot needs to be done to make the manuscript up to standard for publication. They also need to do better in their discussion of the results and it should be done systematically from one result to another in a well-focused manner.

Response: unsure how response associates with the prompt. We have amended the discussion to include additional narriative regarding race adjustment of eGFR for CKD-Epi and MDRD-4 equations.

Figures/ tables are clearly presented and correctly labelled
Methods are detailed enough for another researcher can understand
Statistics are sound enough or further analysis is needed
Designs are appropriate for the question being asked or is there need for additional experiments Are results supporting conclusions and are the data available?
References are missing and the title appropriate for the work done and informative.
Number comments and include page numbers.

MAJOR COMMENTS
They need to clarify how they estimate kidney failure in patients initiating treatment. Also explain if these patients were hospitalized and how they were followed up to determine Kidney Failure. They mentioned a number of endpoints and it made it unclear which one was their primary that they used for powering the study. There was kidney failure in the abstract, there was eGFR<60mL/min/1.73m2 and also moderate kidney dysfunction. How they defined kidney failure needed to come out clearly and at what time points they attempted to observe it.

Response: We have revised and standardized the narrative throughout to "kidney function impairment" which we have set as eGFR<60mL/min/1.73m2 (CKD-Epi equation). Kidney failure is a category of eGFR <15ml/min/m^2.

They need to describe what they used as comparison group in this case and how long after the patient initiated therapy that they had their planned kidney function assessments.

Response: We have amended methods to include additional information about the study model.

They need to explain how many times those with repeated measurements had these measurements done to warrant the use of mixed effect Poisson methods and how many had more than two repeated measurements. Was there a specific follow up time? if not then others were followed after being treated for longer than others which increased their chances of dysfunction due to concomitant

exposure to therapy. Also, there are chances of missing the outcome as the biomarkers stabilize after a long period from injury.

Response: We have included a supplemental table two to outline the potential difference in population between those with single and multiple measures. We have also included detail on the follow-up period for multiple measures, limited to two years.

Since they included anyone with a baseline creatinine, and their inferences made on the entire population, they have to explain how they handled the missing follow up results for more than 90% (65000)of their participants.

Response: We have included supplemental table one to provide insight as to the parent population.

With the very large sample size, where the observed difference clinically significant? A large sample size like this can show statistical significance that is not clinically significant. It was unclear why they opted for a complete enumeration when they had 3209 with repeated measurements from which they could have randomly sampled their study population and avoided all the missing data. Increasing the sample size may not lead to a different conclusion for a research but it may increase the precision.

Response: We have included supplemental table two to better understand any differences between the population with multiple measures and those with baseline only .

There were many missing observations from line (236) of follow up visits more than 90% that was not explained how it was handled. They needlessly attempted to use a very large sample size that gave not extra new information. Mixed methods would be more effective for repeated measure usually more than 2 measures to model the trajectory of an outcome. Logistic regression, cox regression etc would have been better here. Proportional odds ordinal regression for the ordinal outcome on severity of kidney dysfunction.

Response: We have omitted analysis including body mass index, which had extensive missingness though these results are still available in supplemental table three.

They needed to focus their objectives; it appeared that they were interested in the predictors of eGR< 60; to finding the trajectory of the eGFR and method comparisons for eGFR formulae. These needed to be tied in well and focused. Respecting the method comparison, what was the reference method that gave the target eGFR?

Response: We have revised methods to specify that the standard was unadjusted CKD-Epi equation "We conducted mixed-effects Poisson regression on eGFR <60ml/min per unadjusted CKD-EPI formula at baseline (ART initiation) without adjustment for race/ethnicity allowing random effects at facility level"

They need to clarify their inclusion and exclusion criteria and justify that. e.g. did they include those with even those with previous kidney disorders? The enrollment process needs to be more elaborate.

Response: We have provided eligibility criteria in the methods section in the subsection "population".

They need a scientific or clinical basis for categorizing the variables such as age, BMI and blood pressure as they did. The arbitrary categorization which may not be linked to the clinical outcome are problematic.

Response: Body mass index and blood pressure are well specified. Age categorization is consistent with other work in the HIV literature.

They statistical analysis needs to be revisited or properly justified. They need to clarify whether the assumptions for using the parametric tests were met e.g. was eGFR normally distributed for t-test to be used? t-test, chi-square and mixed effects Poisson. They did not do any model diagnostics and validations to show the AUC, PPV, NPV, sensitivity and specificity of their model. Did not explain well in their methods how they came up with the predictors included in the model. They referred to univariate comparisons to make their inferences without adjusting for confounders.

Response: We presented both crude and adjusted estimates in table two.

Tables and figures had to follow. Tables showed be labeled on top and figures below. The information in the table showed be described right above. No key to show what statistical methods were used for the p-values. They did comparisons in the tables among predictors instead of outcomes. Then table 1 and 2 could be condensed into one table.

Response: We have added p-value test detail for table one in the table one footnote. We respectfully disagree that table one and table two should be condensed.

They mentioned diabetes as one of the covariates but there were no observations for this variable to include in the model (99.99 missing information.

Response: We reviewed diabetes and found data to be lacking and was subsequently not included in further analyses.

MINOR COMMENTS

The subheadings for the results (line 174) can be put into one paragraph and the tables 1 and 2 can make one table that compares the different independent variables in relation to the outcome i.e. comparing independent variables among those who developed and those who did not develop the outcome. A well labelled table 1 with a key showing appropriate statistical methods used can be made.

Response: Unclear what the suggestion is here.

A second table can be made from line 236 and address the change in eGFR. This can be to compare the baseline to after therapy and compare among the many dependent variables that can explain the change from baseline. Not comparing independent variables among themselves as in line 240.

Response: Because data were scant among those with multiple measures and our primary aim was to evaluate eGFR at baseline we will refrain from further analyses among those with multiple eGFR measures.

CIDRZ

for a healthy Zambia

It was not clear why the comparisons of the methods from line 263 to 273 were necessary. Why was this being done? These methods already have known differences and applications. They are not bound to give the same results in the first place

Response: This was to describe the heterogeneity in estaimtes and population proportions categorically given the formula applied.

Lines 282-4 does not seem to be well backed by the evidence in the tables. It also did not show whether that was significant or whether that was from regression. The same is true about line 291 to 295.

Response: Unclear how to respond to this comment.

Line 300 the Percents showed be presented with frequencies. e.g. 1/10 is (10%) and so is (100/1000). The discussion from line 313 to 332 is not focused on the findings from the study or the data.

Response: We discuss the possible relationships between CD4 cell count and kidney function as we observed an association between the two in table two.

Recommendations in 341 and 442 are not supported by evidence from data in this paper
347 to 354 Missing data has a lot of chances to cause bias and wrong conclusions especially were the nature of the missingness is linked to the outcome or not by chance. With so much missing data it is not easy do rely on the findings and conclusions. And how do you use Poisson mixed effect models on cross sectional data observed just at baseline and no explanation of what happened to the missing observation and how they were addressed in the study.

Response: Differentiated service delivery is recognized method to improve care and retention among those in HIV care in sub-Saharan Africa

This is true for the entire conclusion section in lines 357 to 361.

Response: Unclear how to respond to this comment.

End of itemized responses

Many thanks again to the reviewers and editorial staff.

Kind Regards,

Jake M. Pry, PhD, MPH
Centre for Infectious Disease Resaerch (CIDRZ)
jmpry@ucdavis.edu | jake.pry@cidrz.org
